# PFAPA and 12 Common MEFV Gene Mutations Our Clinical Experience

**Published:** 2013-11-27

**Authors:** Farhad Salehzadeh, Maryam Vahedi, Saeid Hosseini-Asl, Sepideh Jahangiri, Shahram Habibzadeh, Mahsa Hosseini-Khotbesara

**Affiliations:** Department of Pediatric, Ardabil University of Medical Sciences, Ardabil, Iran

**Keywords:** PFAPA, Gaslini Score, MEFV Gene, MEFV Gene Mutations

## Abstract

***Objective:*** Marshall Syndrome or PFAPA is an inflammatory periodic disease characterized by periodic fever, aphthous stomatitis, pharyngitis and cervical adenitis. Although PFAPA is an auto inflammatory disease, it doesn't have genetic basis such as other periodic fevers. This study evaluates the 12 common MEFV gene mutations in patients with PFAPA syndrome.

***Methods:*** 21 patients with PFAPA syndrome who had diagnostic criteria were enrolled in this study and 12 common MEFV gene mutations i.e. P369S, F479L, M680I (G/C), M680I (G/A), I692del, M694V, M694I, K695R, V726A, A744S, R761H, E148Q evaluated. All the patients were screened for MEFV gene mutations by a reverse hybridization assay (FMF Strip Assay, Vienna lab, Vienna, Austria) according to the instructions provided by the manufacturer.

***Findings*** ***:*** The age of patients was between 6 months to 14 years, and 15 were males. Seven patients had heterozygote and one had compound heterozygote (K695R, V725A) mutation. There were 4 alleles M694V, 3 alleles V726A, 1 allele E148Q and 1 allele K694R. No significant difference existed between mutated patients with non-mutated in symptoms like aphthous and stomatitis, duration of attacks, episodes of fever and response to treatment. Gaslini score test was not helpful to predict the probability of gene mutations.

***Conclusion:*** About 30 percent of patients had MEFV gene mutations but these mutations did not play a main role in presentation of PFAPA symptoms.

## Introduction

Marshall Syndrome was first defined by Marshal in 1987^[^^1^^]^. The acronym FAPA (fever, aphthous stomatitis, pharyngitis, cervical adenitis) was was later renamed to PFAPA (periodic fever, aphthous stomatitis, pharyngitis, and cervical adenitis)^[^^2^^]^. This clinical syndrome occurs among children less than 5 years old. It is a sporadic disease with periodic attacks of inflammation^[^^3^^]^. Periodic fevers usually last 3 to 6 days and occur with regular intervals about every 3 to 6 weeks. The diagnosis is established on the basis of clinical criteria that require the presence of a recurrent fever of early onset (<5 years) and ≥1 of the 3 associated symptoms (aphthosis, cervical adenitis, and pharyngitis) in the absence of upper respiratory tract infections and cyclic neutropenia^[^^2^^]^.

 Between episodes, the children usually are well. 

The exact cause of PFAPA is unknown and autoimmune or infectious processes in its pathogenesis are not proven. Moreover no geographical or ethnic tendencies have been shown. WBC and ESR are increased during acute episodes^[^^4^^]^. Because a single dose of corticosteroid can resolve attacks, inflammatory cytokines production in response to infectious factors is considered as a leading cause of the syndrome. IFN-γ, TNF and IL-6 increase during episodes^[^^5^^,^^6^^]^. It is more common in males and the prognosis is very good^[^^7^^,^^8^^]^. 

 PFAPA has been reported in adult patients too^[^^9^^,^^10^^]^. Although PFAPA is a periodic fever, no specific gene mutation is known for it. Nevertheless it is possible that genes involved in other inflammatory diseases play a role in its pathogenesis^[^^2^^]^. 

 In Cazeneuve’s study among 12 allele analysis only one mutation of M694V was found^[^^11^^]^. Berkun study showed that in PFAPA patients with MEFV gene mutation the disease was less severe and aphthous stomatitis, duration of disease, number of attacks, and need to steroid were less common. Therefore they found MEFV gene effect as a modifier on PFAPA^[^^12^^]^. 

 In a study on 393 children with PFAPA, the clinical features were compared in genetically negative and positive results, 82 had positive genetic results, 75 uncertain and 236 negative results. Jn patients with positive genetic results diarrhea, vomiting, arthralgia and rash was more frequent. In the genetically negative group exudative pharyngitis was encountered more frequently^[^^13^^]^.

 Gattorno et al evaluated the relationship between PFAPA and other periodic fevers such as FMF, MVK, and TRAPS. They found a close relationship between PFAPA and other periodic fever syndromes^[^^14^^]^.

 FMF is an autosomal recessive disease characterized by acute attacks of fever and polyserositis. This disease mainly occurs in Mediterranean population and is caused by MEFV gene mutations^[2]^. This study was designed to analyse 12 most common MEFV gene mutations in PFAPA patients and also evaluating Gaslini score test based on detected mutations.

## Subjects and Methods

Twenty one patients who had diagnostic criteria were enrolled in this study. Twelve common MEFV gene mutations were evaluated: P369S, F479L, M680I(G/C), M680I(G/A), I692del, M694V, M694I, K695R, V726A, A744S, R761H, E148Q. All the patients were screened for 12 common MEFV mutations by a reverse hybridization assay (FMF StripAssay, Vienna lab, Vienna, Austria) according to the instructions provided by the manufacturer. Statistical analysis was performed using SPSS 16.0. Comparison between the different genotypes was assessed using chi-square test. A *P*-value <0.05 was accepted as statistically significant.

## Findings

The age of patients was between 6 months and 14 years, and 15 were males. The mean age at onset of symptoms was 1.52±2.72 years (range 6 months - 5 years). The mean duration of fever was 3.57±1.64 days, and that of the disease 4.23±1.56 days. The interval of the attacks was 32.52±20.53 days. All of the patients had fever and 12 (57.14%) patients had abdominal pain, 19 (90.47%) children were breast fed infants.

 In family history one patient's brother, one patient's father and one patient's mother had FMF. Eight (38.09%) patients had MEFV gene mutations, seven of them were heterozygotes and one was combined heterozygous (K695R, V725A). 

(Table 1).

 Although abdominal pain, aphthous stomatitis and duration of attacks were more common in non-mutated patients, these findings in association with episodes of fever and response to treatment did not show meaningful difference between the two groups (*P*≤0.05).

**Table 1:** Prevalence of MEFV gene mutations

**MEFV Gene**	**Prevalence**	**Percent**
**M694V**	4	45
**V726A**	3	33
**K694R**	1	11
**E148Q**	1	11

**Table 2:** Clinical findings in different studies

**Signs and symptoms**	**Our study** **%**	**Gattorno** ^[^^14^^]^ **%**	**Stojanov** ^[^^15^^]^ **%**	**Padeh** ^[^^8^^]^ **%**	**Dagan[** ^16^^]^ **%**
**Fever**	100	--	78	100	--
**Weakness and lethargy**	71.42	--	--	100	--
**Skin rash**	14.28	24	18	--	--
**Headache**	28.6	46	6.5	17.8	--
**Oral aphthosis**	47.61	63	13	67.8	33.3
**Abdominal pain**	57.14	68	42	17.8	35.1
**Myalgia**	61.9	3.5	--	--	--
**Diarrhea**	--	36	--	--	--
**Vomiting**	--	41	--	--	--
**Arthralgia**	13	49	--	10.7	--

## Discussion

Padeh^[^^8^^]^, Stojanov ^[^^15^^]^ and Dagan ^[^^16^^]^ studies in comparison with our results are shown in Table 2. Table 3 shows our results and those of Gattornos.

 Bonyadi revealed that the carrier rate of five common MEFV genes in the Azeri Turkish population was 25.5%, with E148Q being the most common (11.5%) mutation followed by V726A (1.75%). This study indicates that the FMF carrier rate and E148Q mutation frequency are high in the Iranian Azeri Turkish population^[^^17^^]^ and one of our studies showed the frequency of E148Q mutation in FMF patients to be 9.95% (in press). It does not seem that these MEFV mutations in PFAPA patients are related to normal population varieties.

 The authors have known two siblings suffering from FMF with M694V – V726A gene mutation, and PFAPA syndrome, offspring of whom one had monozygotic mutation M694V and the other monozygotic mutation V726A.

 In other authors’ experience there were 2 patients who had FMF and PFAPA simultaneously, it was observed that the first patient had R761H-M694I combined mutations and the second one M680I monozygotic mutation.

 It seems that there is a genetic similarity, on the basis of pathogenesis, between FMF and PFAPA. This genetic similarity has led to suggest a scoring system in genetic analysis of periodic fever syndromes in PFAPA patients^[^^13^^]^.

**Table 3:** MEFV genes mutations in two studies

**Heterozygote**		**Our study**	**Gattorno(14)**
		**No of Mutation= 8**	**No of Mutation= 40**
**Compound ** **Heterozygote **	Number	1	7
	Type of Mutation	K695R/ V726A	M694V/V726A M680I/V726A M694V/E148Q L110P/L110P V726A/E148Q V726A/S108R P369S/R408Q
**Heterozygote**	Number	7	33
	Type of Mutation	M694V (4) V726A (2) E148Q (1)	E148Q (11) K695R (5) M694V (4) A744S (3) V726A (2) P369S (1) V487M (1) R408Q (1) M680I (1) R717L (1)

**Table 4:** Gaslini score in our patients

**No**	**Age**	**Sex**	**Age at ** **Onset**	**Abd ** **pain**	**Diarrhea**	**Chest ** **pain**	**Aphtous** **stomatitis**	**Genotype**	**Family ** **history**	**Gaslini ** **score**
1	5	Boy	2y	Yes	No	No	No	Wt- Wt	No	-0.027
2	7	Boy	3y	Yes	Yes	No	Yes	Wt-M694V	No	+1.38
3	5	Boy	2y	No	No	No	No	Wt- Wt	No	-1.608
4	13	Girl	6 mo	Yes	No	No	Yes	Wt- Wt	No	+1.082
5	6	Boy	4y	Yes	No	No	No	Wt- Wt	No	-0.228
6	7	Boy	6y	Yes	No	No	Yes	Wt- Wt	No	-3.34
7	4	Girl	2y	Yes	No	No	Yes	Wt- Wt	No	-0.124
8	5	Boy	3y	No	No	No	No	Wt- Wt	No	-2.412
9	5	Girl	1y	No	No	No	Yes	Wt-V726A	Father: FMF	-0.802
10	5	Boy	10 mo	No	No	No	No	Wt- Wt	No	-0.67
11	12	Boy	2y	No	No	No	Yes	Wt- Wt	No	-3.112
12	12	Boy	3y	No	No	No	No	Wt-M694V	No	-2.412
13	12	Boy	2y	Yes	No	No	Yes	Wt- Wt	Brother: Periodic Fever	+1.379
14	4	Boy	6 mo	Yes	No	No	Yes	Wt- Wt	No	+1.082
15	14	Girl	6y	Yes	No	No	No	Wt-E148Q	No	-1.836
16	9	Boy	3y	No	No	No	Yes	Wt- Wt	No	-3.916
17	10	Boy	5y	Yes	No	No	Yes	Wt-M694V	No	-2.536
18	13	Boy	5y	No	No	No	Yes	K695R-V726A	No	-5.524
19	8	Boy	2y	No	No	No	Yes	Wt-M694V	No	-3.112
20	7	Girl	3y	Yes	No	No	Yes	Wt-V726A	Mother: FMF	+0.575
21	8	Girl	3y	Yes	No	No	Yes	Wt- Wt	No	-0.928

This scoring is based on clinical criteria and is called Gaslini diagnostic score. If a PFAPA patient has scored above 1.32, he/she will be in a high risk of having others periodic fever syndromes.

 In Gattorno study it was observed that Gaslini diagnostic score is a useful diagnostic tool in evaluating differential diagnosis of PFAPA syndrome.

 Because 38 percent of PFAPA patients in this study had MEFV gene mutations, it seems that genetic basis of FMF has a role in pathogenesis of PFAPA; however there is no significant difference between mutation positive and negative patients in duration of disease, episodes of disease and response to treatment. Table 4 shows patients’ Gaslini diagnostic scoring test.

## Conclusion

This scoring was not helpful to predict the probability of MEFV gene mutation (12 common mutations) in our study and in these patients it has also been shown that MEFV gene mutation doesn't have any specific effect in patient’s clinical manifestations. We should find another way to predict the presence of MEFV gene mutations in this syndrome.
